# Moving in and Out: Dispersion of Cells in Self-Generated Gradients

**DOI:** 10.4172/2155-9899.1000507

**Published:** 2017-05-29

**Authors:** Christina H. Stuelten

**Affiliations:** National Institutes of Health, National Cancer Institute, Laboratory of Cellular and Molecular Biology, 37 Convent Drive, Bethesda, MD 20892, USA

**Keywords:** Self-generated gradient, Directed cell migration, Chemotaxis, Lysophosphatidic acid, LPA, Epidermal growth factor, EGF, Stromal derived factor-1, SDF-1

## Abstract

Migrating cells can influence the direction of their own migration by metabolizing chemoattractants present in their environment. This is illustrated by the dispersal of melanoma cells, which break down lysophosphatidic acid and generate a gradient with increasing concentrations of lysophosphatidic acid distant from the tumor. Melanoma cells can then disperse away from the tumor as they migrate in the self-generated lysophosphatidic acid gradient. Thus, dispersal of tumor cells during invasion of the surrounding stroma might be driven by chemotaxis of cells along self-generated chemoattractant gradients.

## Introduction

Cell motility is necessary for organismic development, maintenance, and survival. The earliest form of directed cell migration was perhaps the migration of single cells towards nutrients [1], and evolved into the highly regulated directed cell migration observed in complex physiological contexts such as development and tissue repair in multicellular organisms. For example, directed migration is crucial for formation of fruiting bodies in the social amoeba Dictyostelium [2,3], and primordial germ cell migration in Drosophila, zebrafish, and mouse [4,5].

During directed migration cells are guided by chemoattractants or chemorepellants present in their environment. Repellent guidance, which leads to migration of cells away from a repellant guidance molecule, has been described mostly in the context of guidance of the nerval growth cone, where repellent guidance molecules include semaphorins, netrins and Slit [6–8]. Chemoattraction-the movement of cells towards a chemoattractant-is observed when cells converge to a site, e.g. during wound closure [9,10] or when immune cells migrate to a site of inflammation [11,12]. Interestingly, migrating cells can also direct their own migration by building chemoattractant gradients and chemotaxing in these self-generated gradients. An example is the migration of the zebrafish lateral line primordium along a self-generated SDF-1/CXCL12 gradient, where the atypical receptor CXCR7 acts as a decoy for CXCL12. Expression of CXCR7 in trailing cells of the lateral line leads to sequestration of CXCL12 and as such generates an extracellular CXCL12 gradient [13–17].

Directed cell migration has been co-opted by tumor cells to aid their dispersal during tumor invasion, and Semaphorin A3 has been shown to promote glioblastoma dispersal [18]. On the other hand, chemoattraction of melanoma cells in self-generated lysophosphatidic acid (LPA) gradients has been shown to aid melanoma cell dispersal [19]. In this melanoma model, cells break down LPA, leading to low LPA levels within or in the immediate vicinity of tumors or cell colonies, compared to higher levels at the tumor edge and surrounding tumor stroma [19]. Thus, the tumor or melanoma cell colony acts as a sink for LPA, while the stroma surrounding the tumor or cells acts as a source. Chemotaxis along this outwards oriented, self-generated LPA gradient allows melanoma cells to migrate away from the tumor and to invade and disperse into the surrounding stroma in a LPA-dependent manner [19]. Chemotaxis along a self-generated gradient of EGF has also been observed in human lung adenocarcinoma cells (PC9), human breast cancer cells (MDA-MB-231), and human mammary epithelial cells [20]. In this case, EGF is removed from the extracellular environment by uptake of EGF into cells, which allows chemotaxis along the resulting gradient. Thus, cells can build self-generated gradients by removing chemoattractants from their environment using at least three mechanisms: (a) binding to decoy receptors, (b) uptake of chemoattractants into cells, and (c) by degradation of chemoattractants.

The concept of chemotaxis along self-generated gradients has several interesting implications. First, migration along self-generated gradients allows cells to chemotax through areas where a chemoattractant is widely expressed. Ubiquitously expressed chemoattractants may therefore be candidates for chemoattractants that act in self-generated gradients.

Second, the steepness of the gradient depends on the capacity of the tumor cells to remove a ubiquitously expressed mediator from their environment. By doing so, tumor cells can migrate away from the original tumor once it reaches a critical mass that allows a sufficiently steep gradient to be formed. As cells migrate away from the tumor the leading cells will keep removing chemoattractant, and one would expect to see an invasive front where cells are exposed to a steep chemoattractant gradient, followed by a ‘trailing end’ where the gradient is shallow and fewer cells migrate with poor directionality. Indeed, it was observed that melanoma cells migrating in a self-generated LPA gradient form a front which is followed by sparser, less oriented cells [19,21]. One would also expect the size of migrating cell clusters to become smaller as the cells disperse, resulting in lesser chemoattractant removal and shallower gradients, until cells come to a stop. These new cell colonies should start migrating again as tumor cells divide and the “satellite colonies” reach a size sufficient to reduce local chemoattractant levels and to generate a relevant gradient. Thus, invasion should occur in waves, where cells proliferate until a gradient sufficient to induce chemotaxis is built, disperse along the gradient until it collapses, and then increase colony size by proliferation until a critical mass is obtained that again allows formation of a self-generated gradient.

Third, self-generated gradients can, in principle, be sustained over long distances and long time periods. As it is the migrating cells themselves that build a gradient necessary for chemotaxis by decreasing the chemoattractant concentration present in the microenvironment, the cells should be able to maintain or re-establish the gradient indefinitely if (a) the chemoattractant is present in the environment and (b) the cells maintain their capacity to reduce the chemoattractant level in their vicinity. This is of importance as tumor cells invade and migrate far into the surrounding stroma in the course of weeks and months.

As cells migrate through the stroma, they may, however, also be exposed to and respond to local gradients of chemoattractants and chemorepellants. Thus, although tumor cells chemotaxing in a self-generated gradient should disperse in a centrifugal manner, the generation of complex self-generated gradients by tumor cell colonies of changing size and the migrating cells encountering other gradients may lead to more complex cell trajectories that result from the integration of multiple competing gradients or guidance cues a cell is exposed to [22]. The effect of the microenvironment and cellular context on the migratory phenotype can be illustrated by the response of melanoma cells to LPA. In the work described above, LPA signaling *via* LPA receptor 1 (LPA1) and degradation of LPA by tumor cells allows the cells to chemotax in a self-generated LPA gradient away from the tumor [19]. In contrast, LPA acts as a repellant guidance clue for mouse melanoma cells (B16) [23]. In this case, LPA mediates its effects *via* a different receptor, LPA5, to inhibit cell migration and to act as a chemorepellant [23]. Thus, LPA5-expressing melanoma cells would only disperse away from a tumor, if LPA levels in the tumor were higher than in the surrounding stroma. This complex regulation of effects of LPA, which depends on the capacity of cells as well as the cells’ environment to produce and remove LPA as well as the cells’ receptor expression profile, illustrates the complex regulation of directed migration by multilayered, non-redundant signaling networks such as the LPA signaling network [19,23–25].

In conclusion, self-generated gradients can be established by removal of chemoattractants from the microenvironment by degradation, uptake, or binding to decoy receptors. These self-generated gradients as they are observed in dispersing melanoma cells facilitate long-distance and long-term dispersal of cells through tissues as is observed during tumor cell invasion and possibly during reverse interstitial immune cell migration. However, these gradients and the response of cells to self-generated gradients might be modulated by other guidance cues existing in a cell’s environment.
